# Comparison of the Catquest-9SF and Cat-PROM5 Patient-Reported Outcome Measures: A Literature Review

**DOI:** 10.7759/cureus.94743

**Published:** 2025-10-16

**Authors:** Jesse M Britt, Austin C Ellyson

**Affiliations:** 1 Aviation Medicine, Lyster Army Health Clinic, Fort Rucker, USA; 2 Ophthalmology, Madigan Army Medical Center, Tacoma, USA

**Keywords:** cataract, cat-prom5, catquest-9sf, patient-reported outcome measures, questionnaire, survey

## Abstract

Patient-reported outcome measures (PROMs) have become increasingly more popular in modern medicine. The use of validated, precise, and succinct PROMs is necessary to accurately assess for changes in patients’ quality of life (QoL) postoperatively and keep pace with high-volume surgery clinics. Cataract surgery is one of the most performed surgeries worldwide. The Catquest-9SF and Cat-PROM5 are PROM tools for assessing QoL improvement in postoperative cataract patients. The aim of this review is to present the advantages and disadvantages of the Catquest-9SF and the Cat-PROM5 and to discuss the clinical relevance of each. A PubMed search was performed without field restrictions for “Catquest-9SF” and “Cat-PROM5”, dated January 2009 to March 14, 2025. This identified 105 articles: 95 of those referenced the Catquest-9SF, 11 of those referenced the Cat-PROM5, and one of those referenced both. These were screened to include randomized control trials, meta-analyses, systematic reviews, clinical studies, comparative studies, reviews, observational studies, and validation studies. This provided a total of 52 studies, eight studies focused on Cat-PROM5, 45 focused on Catquest-9SF, and one study with both. A review of the Cochrane database was also performed that yielded no additional items. Based on data of psychometric properties from the direct comparison study, both PROM tools exhibited excellent performance with Person Reliability indices of 0.90 (Cat-PROM5) and 0.88 (Catquest-9SF) as well as 1.45 SD (Cat-PROM5) and 1.47 SD (Catquest-9SF) for responsiveness to surgery using Cohen’s standardized effect size with a correlation of R=0.85. Both the Catquest-9SF and the Cat-PROM5 perform similarly regarding psychometric properties. However, the Cat-PROM5 has a larger font and fewer questions (five items) that is preferred by patients. In contrast, the Catquest-9SF has more questions (nine items), has more data, is validated in multiple different languages, and is proven to be useful to assess other ocular conditions apart from cataracts. This would suggest that either tool could be used to great effect when assessing postoperative cataract patients, but the Catquest-9SF may be the tool of choice for other ocular conditions or procedures.

## Introduction and background

Cataracts are the leading cause of blindness worldwide and account for half of all blindness in developing countries and 5% in developed countries. As such, cataract surgery is one of the most performed procedures globally [1]. The traditional outcome metric of monocular visual acuity is not perfect at assessing patient improvement post-operatively, and an effort has been made to focus on patient-reported outcome measures (PROMs) to gauge quality of life improvement [2]. In the field of ophthalmology, the two PROMs deemed to be most useful in assessing post-operative cataract patients are the Catquest-9SF, originally developed by Lundström and Pesudovs (2009) [3], and the Cat-PROM5, originally developed by Sparrow et al., (2018) [4] which can be seen in Appendix Figures 2-3 and Appendix Figures 4-9, respectively. Several advantages and disadvantages of Catquest-9SF and Cat-PROM5 exist, but only one study was found that directly compared the two [5]. This review will discuss the potential pros and cons of either PROM tool regarding length, validity, adaptability, and psychometric properties.

## Review

Methods

**Figure 1:**
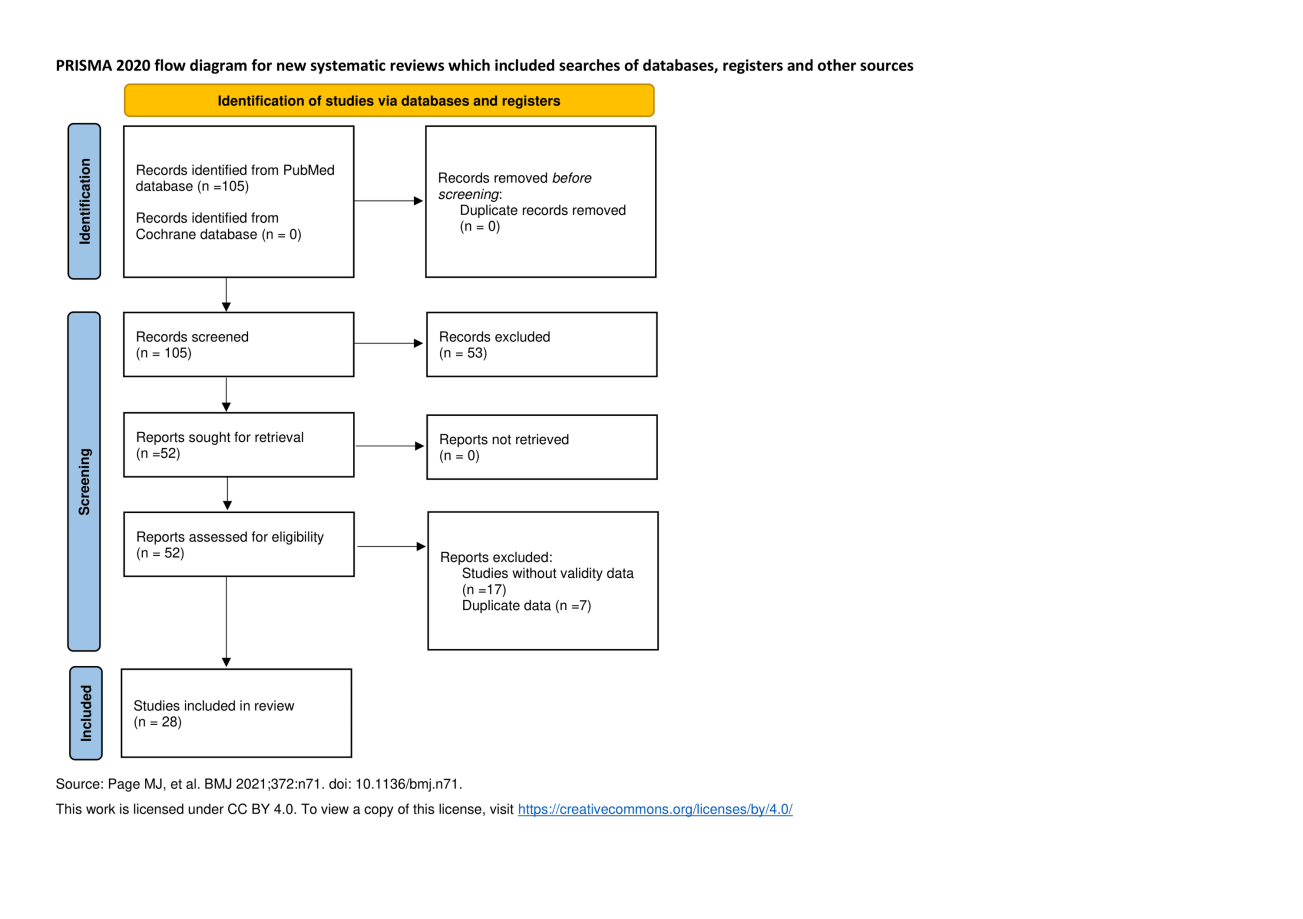
PRISMA 2020 flow diagram for new systematic reviews which included searches of databases, registers and other sources Source: Page et al. (2021) [6]. This work is licensed under CC BY 4.0. To view a copy of this license, visit https://creativecommons.org/licenses/by/4.0/. PRISMA: Preferred Reporting Items for Systematic reviews and Meta-Analyses.

A literature search was performed on PubMed database without field restrictions for search terms of “Catquest-9SF” and “Cat-PROM5”, dated January 2009 to March 14, 2025. A review of Cochrane database was also performed using the same terms on March 14, 2025, that yielded no additional items. Inclusion criteria included randomized control trials, meta-analyses, systematic reviews, clinical studies, comparative studies, reviews, observational studies, and validity studies. Exclusion criteria included articles that did not publish validity data. This identified 105 articles: 95 of those referenced the Catquest-9SF, 11 of those referenced the Cat-PROM5, and one of those referencing both. These were screened by both authors independently for relevance using aforementioned inclusion criteria. This provided a total of 52 studies, eight studies focused on Cat-PROM5, 46 focused on Catquest-9SF, and one study with both. Of these 52 studies, 35 studies were determined to have published validity data with 32 studies evaluating Catquest-9SF, four studies evaluating Cat-PROM5, and one study evaluating both. The remaining 35 articles were then screened for duplicate data. It was determined that seven of those articles had data published in a previous systematic review. These were excluded as the data were accessible in the review and the source articles did not further contribute to the discussion. The remaining 28 articles were then screened for languages each PROM tool was validated in and validation studies outside of traditional postoperative cataract assessment. Given the narrative scope of the study, data from these studies was unaltered and presented as a range. Preferred Reporting Items for Systematic reviews and Meta-Analyses (PRISMA) flowchart is given in Figure 1 [6].

Catquest-9SF

The Catquest-9SF is a nine-item PROM assessment tool that was developed in 2009 by Lundstrom and Pesudovs looking at data collected from 1995 to 2005 from 10,486 questionnaires [3]. Since that time, the Catquest-9SF has been used in over 100 studies and applied to millions of postoperative cataract patients [7]. Despite having nine items, the survey fits on two single-sided sheets or one double-sided sheet of paper with a font size ranging from 12 to 18 (Appendix Figures 2-3). The Caquest-9SF has been validated via Rasch analysis in 12 different languages (Chinese, Danish, Dutch, English, French, German, Italian, Malay, Portuguese, Russian, Spanish, and Swedish) and proven useful when delivered through electronic means [7-9]. The Catquest-9SF has been used in numerous studies and is considered the standard PROM by the International Consortium for Health Outcomes Measurement (ICHOM) for assessing quality of life (QoL) improvement in postoperative cataract patients [10-12]. The Catquest-9SF has also been adapted and used as a PROM for other ocular conditions and interventions such as postoperative corneal transplant or endothelial keratoplasty patients, patients with a diagnosis of Fuchs dystrophy, and post-yttrium aluminum garnet (YAG) laser capsulotomy for posterior capsular opacification (PCO) [13-16]. The Catquest-9SF also repeatably demonstrated excellent psychometric properties in numerous studies [3,5,7,10,17-23].

Cat-PROM5

The Cat-PROM5 is a five-item PROM assessment tool that was developed in 2018 by Sparrow et al. with data collected from 822 participants yielding 1,266 questionnaires demonstrating excellent responsiveness and test-retest reliability with an intra-class correlation coefficient of 0.89 [4]. The Cat-PROM5 has been used in 11 PubMed indexed studies, eight of which are cited here [4,5,24-29]. Given the overall format and larger font size ranging from 14.02-36, the Cat-PROM5 requires six single-sided sheets of paper or three double-sided sheets of paper (Appendix Figures 4-9). The Cat-PROM5 has been validated and directly compared to the Catquest-9SF and found to have similar psychometric properties [5]. In a study by Faneli et al., the Cat-PROM5 was recently translated and validated via Rasch analysis in Brazilian Portuguese [29]. The Cat-PROM5 also demonstrated additional utility for assessing patient QoL measures regarding giant cell arteritis [25].

Results

Comparison

The Catquest-9SF and Cat-PROM5 were compared (Table 1) using some basic metrics to provide a broad overview of the two assessment tools. The Catquest-9SF has been functioning over twice as long as the Cat-PROM5 and as such also has eight times as many validation studies. However, the Cat-PROM5 has nearly half the response items when compared to the Catquest-9SF. Although the Catquest-9SF has more response items, the Cat-PROM5 takes up three times as many pages likely due to the larger font size. Regarding languages, Catquest-9SF has been translated and validated in 12 distinct languages [7] and validated for use on electronic platforms [7-9], whereas the Cat-PROM5 has been translated and validated in English [4] and Portuguese [29]. However, both the Catquest-9SF and the Cat-PROM5 display additional functions aside from assessing postoperative cataract patients with four studies identified for the Catquest-9SF and two for the Cat-PROM5 that were not pertaining to the assessment of pre- and postoperative cataract patients [13-16,25,26]. The psychometrics of the two QoL assessment tools does not differ drastically with Catquest-9SF having a test-retest reliability range of 0.85-0.93 and the Cat-PROM5 having a range of 0.87-0.89 (Table 1). The primary disadvantage of both the Catquest-9SF and the Cat-PROM5 would be design focused around the assessment of postoperative cataract patients. This differs from a more general and comprehensive QoL assessment tool of ocular health and vision impairment, like the National Eye Institute Visual Function Questionnaire 25 (NEI-VFQ-25) [2].

**Table 1:** General information and metrics of Catquest-9SF and Cat-PROM5 *Studies available on PubMed that involved validation via Rasch analysis. **Studies that involved use of the PROM in a setting different from postoperative cataract assessment. ***Data on the Catquest-9SF derived from Lundström and Pesudovs 2009 [3] and subsequent validation studies [7-23]. Data on the Cat-PROM5 derived from Sparrow et al., 2018 [4] and subsequent validation studies [24-29]. PROM: patient-reported outcome measure.

PROM	Years in service	Response items	Length in one-sided pages	Validity studies *	Languages	Adaptability studies **	Test-Retest Reliability ***
Catquest-9SF	16	9	2	32	12	3	0.85-0.93
Cat-PROM5	7	5	6	4	2	2	0.87-0.89

Discussion

The Catquest-9SF has the advantage over the Cat-PROM5 in that it has been functioning for a longer period and thus has more studies supporting it and the endorsement of ICHOM as the “standard” PROM for assessment of QoL improvement in postoperative cataract patients [2,10,11]. However, the Cat-PROM5 has fewer response items with a larger font that was preferred by patients over the Catquest-9SF [5]. Likewise, the age of the Catquest-9SF could be perceived as a disadvantage given that the wording and phrasing of response items such as Item C (Appendix Figures 2-3) “…seeing to do handicrafts, woodwork” can seem a bit dated. This has been reported as a point of confusion for some patients and can seem redundant as “…seeing to engage in an activity/hobby that you are interested in” is an item as well that appears similar. The Catquest-9SF could be improved upon by removing and/or editing some of these items and would likely still achieve excellent psychometric results. When compared to the Catquest-9SF, the Cat-PROM5 takes up more pages which is likely due to overall format and increased font size (Appendix Figures 2-3, Appendix Figures 4-9). In a clinical setting, this presents issues for the Cat-PROM5 with increased printing requirements and potentially longer times for patients to complete. These factors can be important in the high-volume setting of ophthalmology. Similarly, the Cat-PROM5 has not been validated in as many different languages, cultures, or electronic mediums as the Catquest-9SF suggesting that the Catquest-9SF is likely more broadly applicable in most cases. This is not to say that the Cat-PROM5 is not equally useful, but rather that it should be applied and validated in similar context to the Catquest-9SF for a more accurate comparison. The Cat-PROM5 does offer utility outside of the traditional PROM and QoL assessment in the postoperative cataract patient [25,26], and it may perform similarly to Catquest-9SF in assessing QoL change in postoperative corneal transplants and endothelial keratoplasty, post-YAG laser procedure for PCO treatment, and measuring Fuchs dystrophy severity and impact on QoL [13-16]. Regarding test-retest reliability, the person reliability index of Catquest-9SF ranges from 0.85 to 0.93, whereas the person reliability index of Cat-PROM5 ranges from 0.87 to 0.89 (Table 1). As illustrated in Table 1, this range was reported from a list of studies available on PubMed database where Catquest-9SF has 32 validity studies compared to four validity studies for Cat-PROM5.

Future Research

Future studies comparing the Catquest-9SF and Cat-PROM5 should be conducted assessing factors such as ease of use, patient preference, psychometrics, and validity. Studies could be modeled after study done by Sparrow et al. [4]. These QoL assessment tools could also stand to be improved to increase ease of use and patient preference. For example, the Catquest-9SF could reduce the number of response items from nine to five as there is some redundancy. As for the Cat-PROM5, it may benefit from reformatting to allow the survey to fit on fewer pages. Additionally, the two tools could be combined to include the best qualities of each to form a new PROM that theoretically should have comparable psychometric properties and improved ease of use.

## Conclusions

The Catquest-9SF and Cat-PROM5 were compared and determined to have similar metrics. The Catquest-9SF has been functioning over twice as long as the Cat-PROM5 and as such also has eight times as many validation studies. However, the Cat-PROM5 has nearly half the response items when compared to the Catquest-9SF. Although the Catquest-9SF has more response items, the Cat-PROM5 takes up three times as many pages likely due to the larger font size. Regarding languages, Catquest-9SF has been translated and validated in 12 distinct languages and validated for use on electronic platforms, whereas the Cat-PROM5 has been translated and validated in English and Portuguese. However, both the Catquest-9SF and the Cat-PROM5 display additional functions aside from assessing postoperative cataract patients with four studies identified for the Catquest-9SF and two for the Cat-PROM5 that were not pertaining to the assessment of pre- and postoperative cataract patients. The psychometrics of the two QoL assessment tools does not differ drastically with Catquest-9SF having a test-retest reliability range of 0.85-0.93 and the Cat-PROM5 having a range of 0.87-0.89. Ultimately, the choice of which QoL assessment to use should be individualized based on patient population, the goal of the assessment, and provider preference.

## Figures and Tables

**Figure 2:**
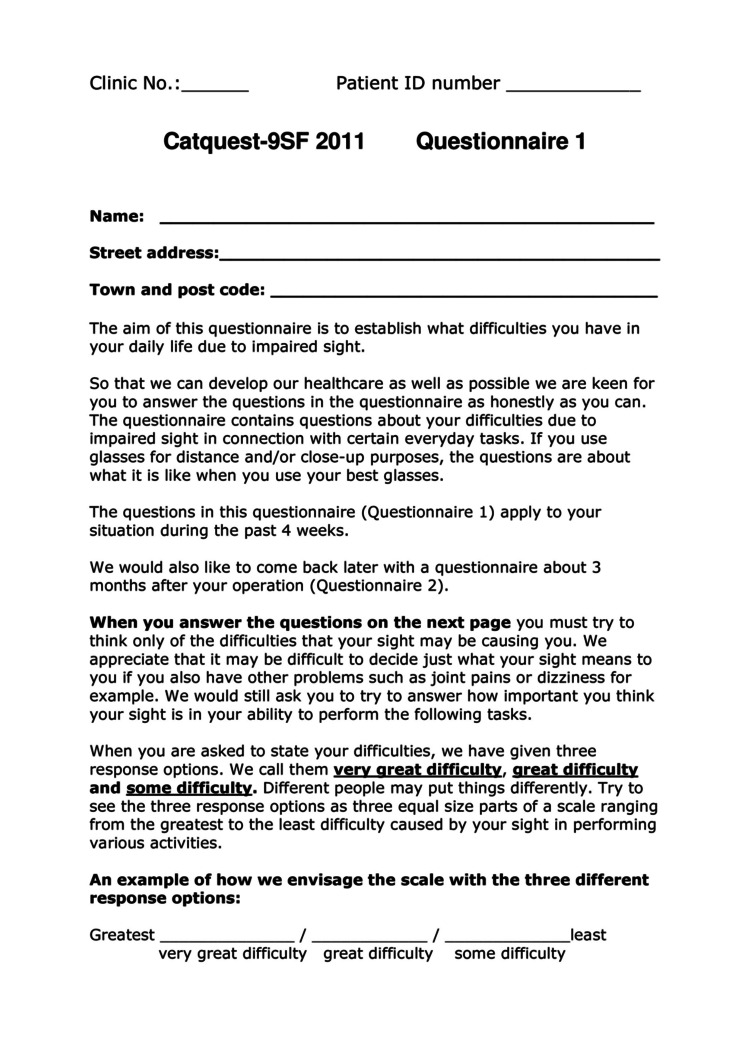
Catquest-9SF survey pg. 1 Reproduced with permission from Lundström and Pesudovs, 2009 [3]. Catquest-9SF is available and open to public and has no licensing requirements.

**Figure 3:**
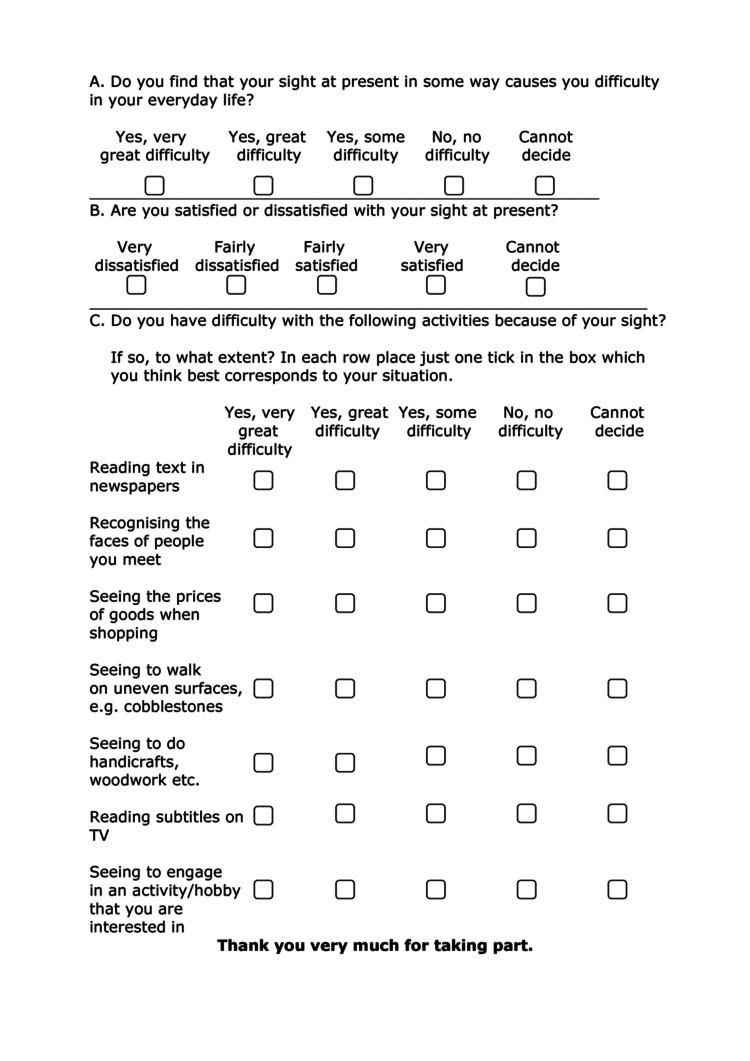
Catquest-9SF pg. 2 Reproduced with permission from Lundström and Pesudovs, 2009 [3]. Catquest-9SF is available and open to public and has no licensing requirements.

**Figure 4:**
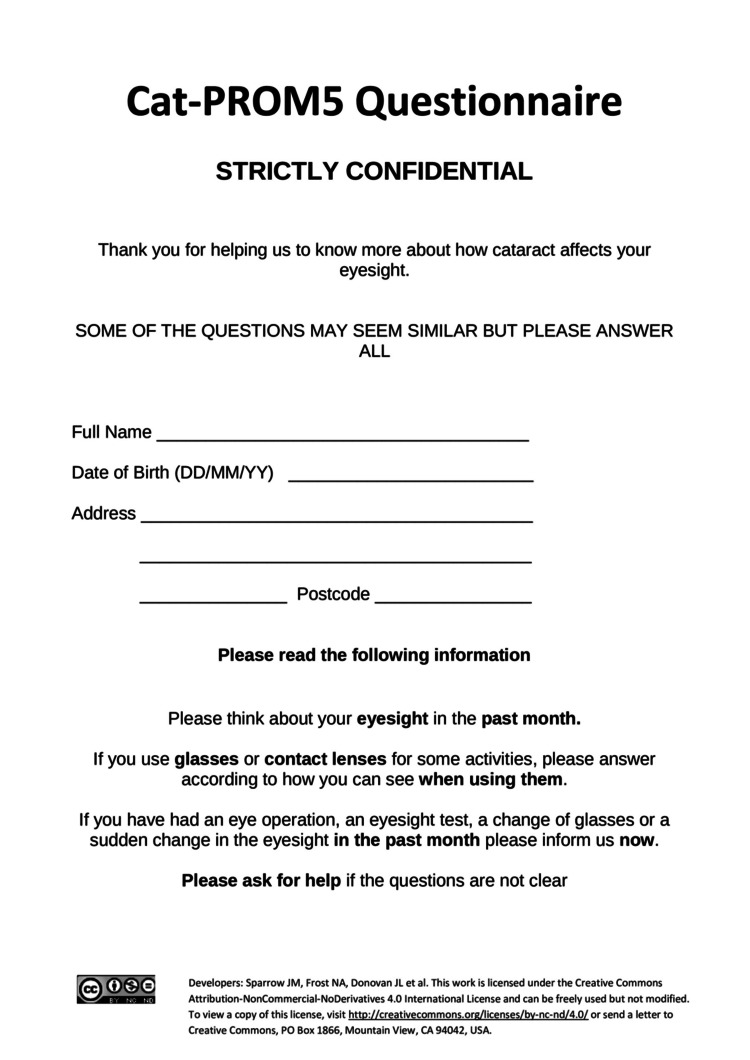
Cat-PROM5 survey pg. 1 Reproduced with permission from Sparrow et al., 2018 [4]. Cat-PROM5 is licensed under Creative Commons but does not require approval outside of commercial use.

**Figure 5:**
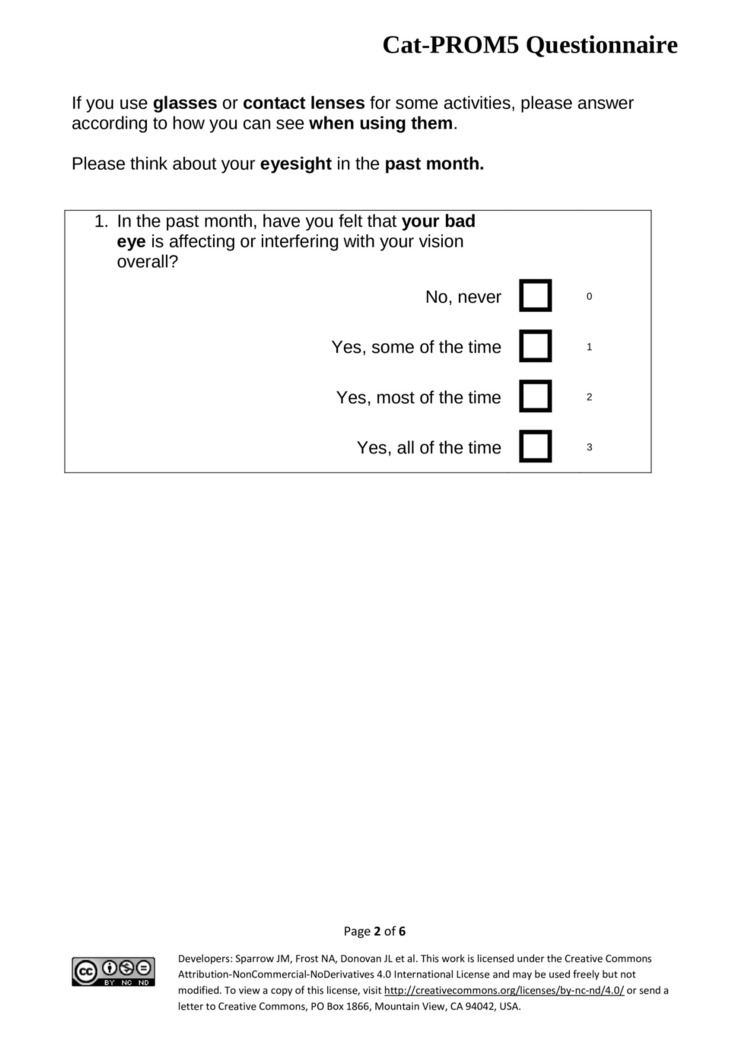
Cat-PROM5 survey pg. 2 Reproduced with permission from Sparrow et al., 2018 [4]. Cat-PROM5 is licensed under Creative Commons but does not require approval outside of commercial use.

**Figure 6:**
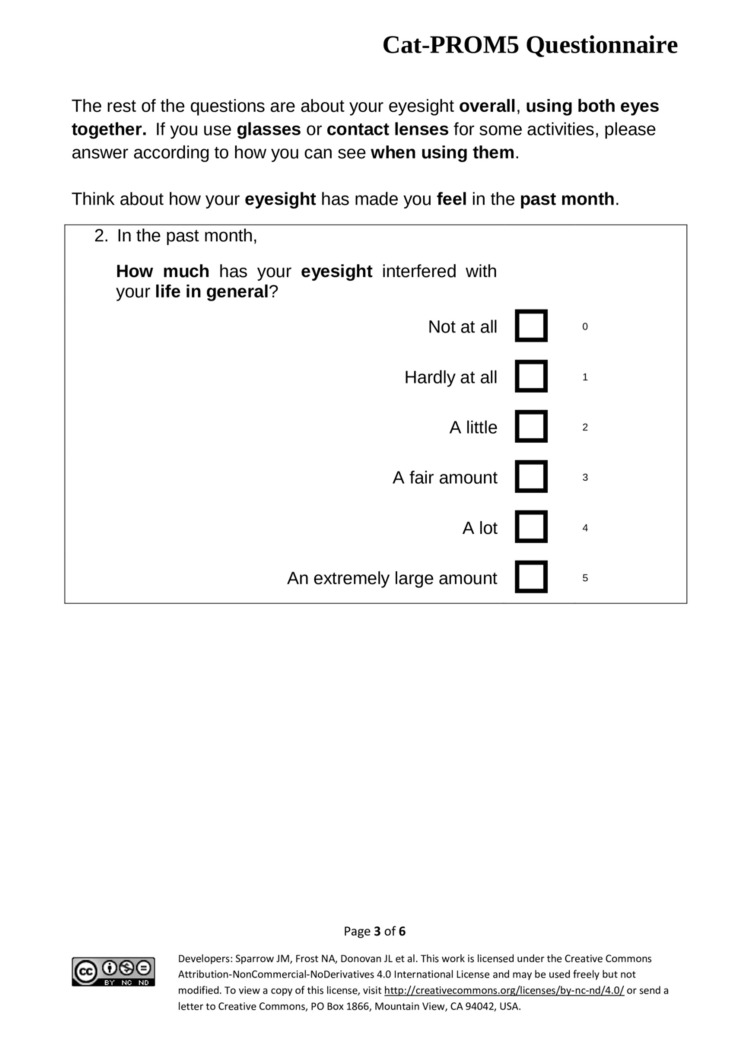
Cat-PROM5 survey pg. 3 Reproduced with permission from Sparrow et al., 2018 [4]. Cat-PROM5 is licensed under Creative Commons but does not require approval outside of commercial use.

**Figure 7:**
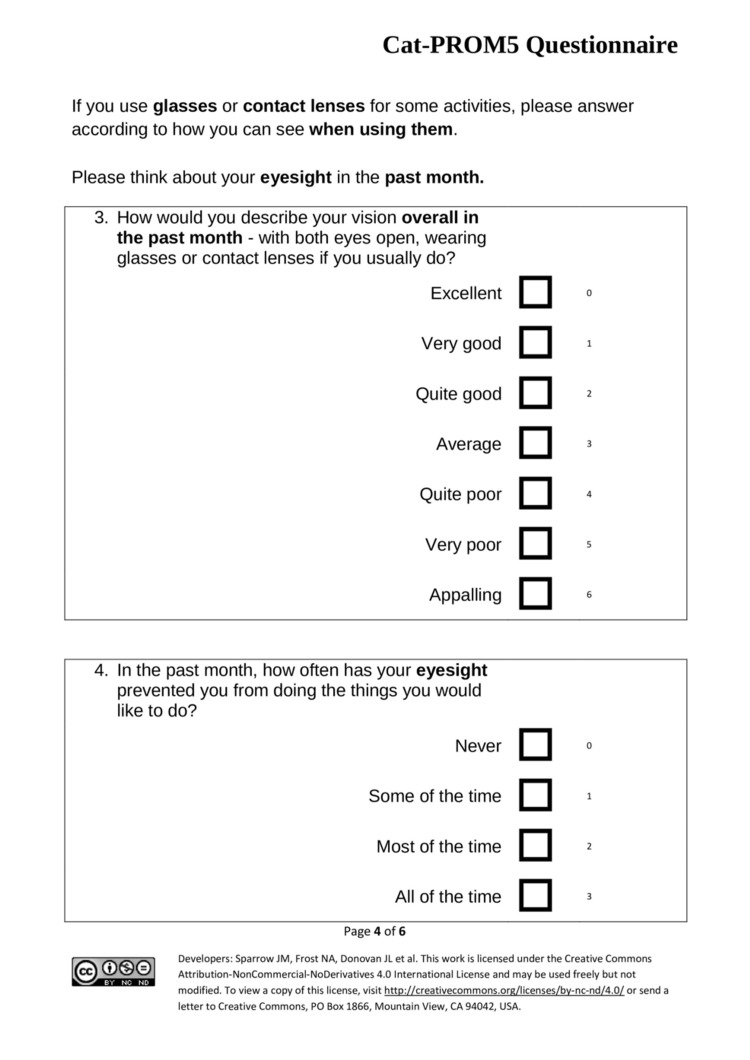
Cat-PROM5 survey pg. 4 Reproduced with permission from Sparrow et al., 2018 [4]. Cat-PROM5 is licensed under Creative Commons but does not require approval outside of commercial use.

**Figure 8:**
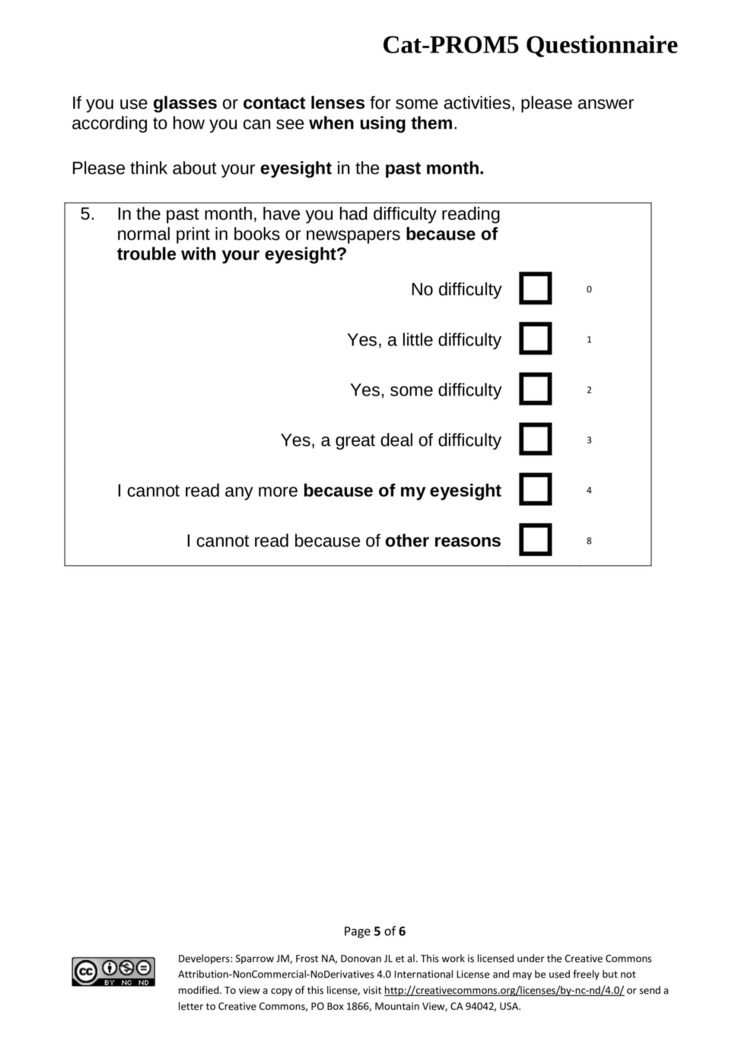
Cat-PROM5 survey pg. 5 Reproduced with permission from Sparrow et al., 2018 [4]. Cat-PROM5 is licensed under Creative Commons but does not require approval outside of commercial use.

**Figure 9:**
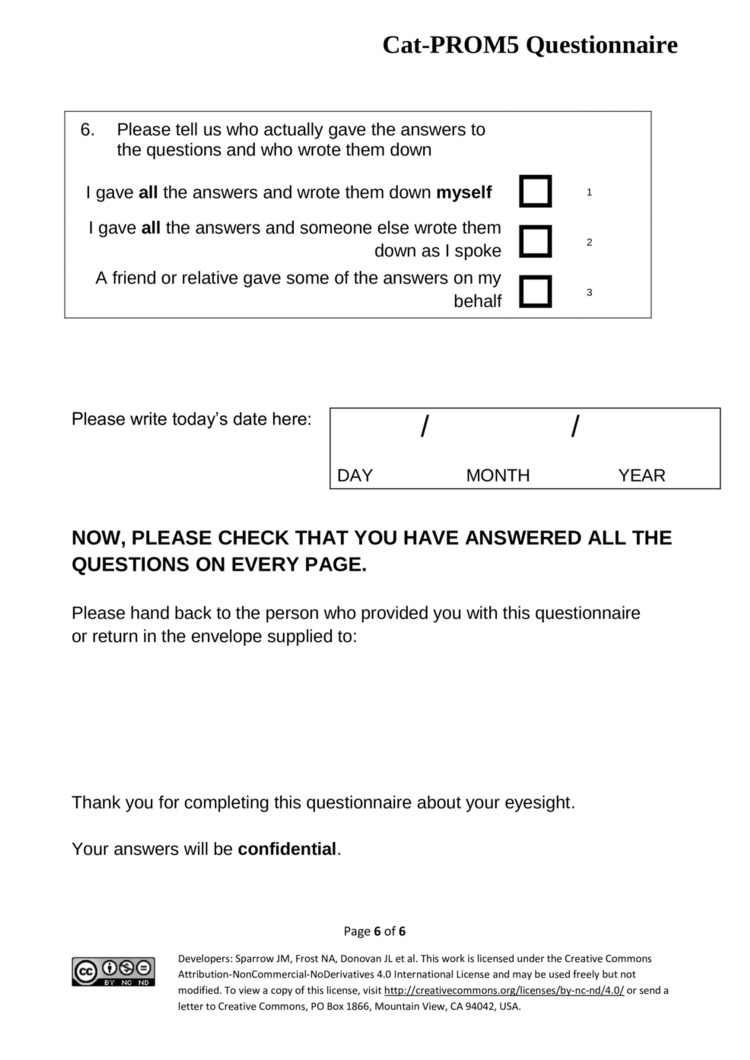
Cat-PROM5 survey pg. 6 Reproduced with permission from Sparrow et al., 2018 [4]. Cat-PROM5 is licensed under Creative Commons but does not require approval outside of commercial use.

## References

[1] Ang MJ, Afshari NA (2021). Cataract and systemic disease: a review. Clin Exp Ophthalmol.

[2] Braithwaite T, Calvert M, Gray A, Pesudovs K, Denniston AK (2019). The use of patient-reported outcome research in modern ophthalmology: impact on clinical trials and routine clinical practice. Patient Relat Outcome Meas.

[3] Lundström M, Pesudovs K (2009). Catquest-9SF patient outcomes questionnaire: nine-item short-form Rasch-scaled revision of the Catquest questionnaire. J Cataract Refract Surg.

[4] Sparrow JM, Grzeda MT, Frost NA (2018). Cat-PROM5: a brief psychometrically robust self-report questionnaire instrument for cataract surgery. Eye (Lond).

[5] Sparrow JM, Grzeda MT, Frost NA (2018). Cataract surgery patient-reported outcome measures: a head-to-head comparison of the psychometric performance and patient acceptability of the Cat-PROM5 and Catquest-9SF self-report questionnaires. Eye (Lond).

[6] Page MJ, McKenzie JE, Bossuyt PM (2021). The PRISMA 2020 statement: an updated guideline for reporting systematic reviews. Br Med J.

[7] Kabanovski A, Hatch W, Chaudhary V, El-Defrawy S, Reid R, Ahmed II, Schlenker MB (2020). Validation and application of Catquest-9SF in various populations: a systematic review. Surv Ophthalmol.

[8] Claessens JL, Wanten JC, Bauer NJ (2024). Web-based telemonitoring of visual function and self-reported postoperative outcomes in cataract care: international multicenter randomized controlled trial. J Cataract Refract Surg.

[9] Lundström M, Kugelberg M, Montan P, Nilsson I, Zetterberg M, Pesudovs K, Behndig A (2020). Catquest-9SF functioning over a decade: a study from the Swedish National Cataract Register. Eye Vis (Lond).

[10] Katz G, Rouquette A, Lignereux F, Mourgues T, Weber M, Lundström M (2021). Validity of the French version of Catquest-9SF and use of an electronic notepad for entering patient-reported outcome measures. Eye Vis (Lond).

[11] Zijlmans BL, van Zijderveld R, Manzulli M (2021). Global multi-site, prospective analysis of cataract surgery outcomes following ICHOM standards: the European CAT-Community. Graefes Arch Clin Exp Ophthalmol.

[12] Tognetto D, Giglio R, De Giacinto C, Dell'Aquila C, Pian G, Scardellato C, Piñero DP (2021). Cataract standard set for outcome measures: an Italian tertiary referral centre experience. Eur J Ophthalmol.

[13] Queirós L, Redondo P, França M (2021). Implementing ICHOM standard set for cataract surgery at IPO-Porto (Portugal): clinical outcomes, quality of life and costs. BMC Ophthalmol.

[14] Wacker K, Baratz KH, Bourne WM, Patel SV (2018). Patient-reported visual disability in Fuchs' endothelial corneal dystrophy measured by the visual function and corneal health status instrument. Ophthalmology.

[15] Claesson M, Armitage WJ, Byström B, Montan P, Samolov B, Stenvi U, Lundström M (2017). Validation of Catquest-9SF: a visual disability instrument to evaluate patient function after corneal transplantation. Cornea.

[16] Nielsen E, Ivarsen A, Kristensen S, Hjortdal J (2016). Fuchs' endothelial corneal dystrophy: a controlled prospective study on visual recovery after endothelial keratoplasty. Acta Ophthalmol.

[17] Sundelin K, Lundström M (2012). Outcome of capsulotomy in patients with low vision and posterior capsule opacification. Acta Ophthalmol.

[18] Bro T, Behndig A, Viberg A (2023). Two point four million cataract surgeries: 30 years with the Swedish National Cataract Register, 1992-2021. J Cataract Refract Surg.

[19] Stolk-Vos AC, Visser MS, Klijn S (2018). Effects of clinical parameters on patient-reported outcome in cataract patients: a multicentre study. Acta Ophthalmol.

[20] Xu Z, Wu S, Li W, Dou Y, Wu Q (2018). The Chinese Catquest-9SF: validation and application in community screenings. BMC Ophthalmol.

[21] Visser MS, Dieleman M, Klijn S, Timman R, Lundström M, Busschbach JJ, Reus NJ (2017). Validation, test-retest reliability and norm scores for the Dutch Catquest-9SF. Acta Ophthalmol.

[22] Fung SS, Luis J, Hussain B, Bunce C, Hingorani M, Hancox J (2016). Patient-reported outcome measuring tools in cataract surgery: clinical comparison at a tertiary hospital. J Cataract Refract Surg.

[23] Lin X, Li M, Wang M (2014). Validation of Catquest-9SF questionnaire in a Chinese cataract population. PLoS One.

[24] Luo Y, Li H, Chen W, Gao Y, Ma T, Ye Z, Li Z (2022). A prospective randomized clinical trial of active-fluidics versus gravity-fluidics system in phacoemulsification for age-related cataract (AGSPC). Ann Med.

[25] Ndosi M, Almeida C, Dawson J (2024). Validation of a patient-reported outcome measure for giant cell arteritis. Rheumatology (Oxford).

[26] Breheny K, Hollingworth W, Kandiyali R (2020). Assessing the construct validity and responsiveness of Preference-Based Measures (PBMs) in cataract surgery patients. Qual Life Res.

[27] Naderi K, Jameel A, Chow I, Hull C, O'Brart D (2024). Effects of axis-flip of the refractive cylinder on vision and patient-reported outcome measures after toric intraocular lens implantation. J Cataract Refract Surg.

[28] Dixon P, Hollingworth W, Sparrow J (2020). Mapping to quality of life and capability measures in cataract surgery patients: from Cat-PROM5 to EQ-5D-3L, EQ-5D-5L, and ICECAP-O using mixture modelling. MDM Policy Pract.

[29] Faneli A, Oliveira R, Marback E, Souza M, Marback P, Laporte L, Muccioli C (2025). Validation of the Brazilian Portuguese version of the cat-PROM5 questionnaire. JFO Open Ophthalmol.

